# A study of polymerase chain reaction device control via cloud using Firebase Cloud Messaging protocol

**DOI:** 10.1186/s12938-018-0585-2

**Published:** 2018-11-06

**Authors:** Jong-Dae Kim, So-Yeon Lee, Yu-Seop Kim, Hye-Jeong Song, Chan-Young Park

**Affiliations:** 1 Department of Convergence Software, Hallym University, 1, Hallymdaehak-gil, Chuncheon, Republic of Korea; 2 Bio-IT Research Center, Hallym University, 1, Hallymdaehak-gil, Chuncheon, Republic of Korea

**Keywords:** Polymerase chain reaction (PCR), Internet of Things (IoT), Cloud computing, Firebase Cloud Messaging (FCM), Realtime monitoring

## Abstract

**Background:**

In this paper, we propose a system for data monitoring and control of polymerase chain reaction (PCR) externally. PCR is a technique for amplifying a desired DNA molecule by repeatedly synthesizing a specific part of DNA sequence. Currently, commercially available systems are standalone systems or operate PCR devices through a computer in the vicinity of devices for control purposes. These systems are limited in the number of devices that the host system can monitor at the same time, and there are limitations in controlling devices or accessing experimental data externally. Therefore, we propose a system to control the PCR device via the cloud for the convenience of the user and to overcome the limitation of the place.

**Methods:**

The cloud system used in this study is Google’s Firebase. At this time, we use Firebase Cloud Messaging (FCM) protocol to send and receive data. In this paper, we have experimented on the possibility of data transmission and reception using FCM between device, cloud and user. Since the PCR chips used in the research are generally operated at about 10°/s, and the temperature can be controlled within 0.5°, the processing period of the control process should be made much smaller than 1/20 s (50 ms).

**Results:**

As a result of experiments, the time of the data round-trip using FCM was measured at 150 ms on the average. Therefore, the data exchange time using FCM is three times slower than the reference time of 50 ms.

**Conclusions:**

Since the data round-trip time using FCM is measured to be three times slower than the reference time of 50 ms, it is impossible for the user to control the device such as the PCR device used in this study through the cloud. However, it is possible for the user to monitor the status of the PCR device from the outside in real time.

## Background

Polymerase chain reaction (PCR) is a molecular biology technique that can diagnose and analyze diseases by replicating or amplifying specific regions of DNA by repeating a series of thermal control reaction steps [1, 2]. DNA can be amplified in a large number of hours in a specific region of a DNA sample by a PCR. This technique is an important skill used to handle DNA and RNA, such as molecular biology, medical care, criminal investigations, and biology. The PCR consists of three stages, such as denaturation, annealing and extension [3]. These three steps are automatically executed if the temperature values and time are set on the PCR device.

Currently, the commercialized system is a standalone system or operates a PCR device through a computer near the control device [4]. This system has problems that can cause inconvenience to the user. First, the number of devices that a host system can simultaneously monitor is limited. The second problem is that there are restrictions on controlling the device from outside or accessing experimental data externally [5]. Third, there is a limitation in the space in which the user must be located near the apparatus during the operation of the PCR device [6]. The fourth is to purchase a computer to control the PCR device to build the environment of the PCR device [7]. Since these problems, the convergence of the PCR technology and the Internet of Things (IoT) technologies is to provide users with greater convenience than using conventional PCR devices. IoT technology is expected to take up a large part of modern life. Thus, the PCR system used in this study was developed applying IoT technology to complement the previously mentioned shortcomings. Also, this system applied cloud computing technology for centralized management of data [8].

Currently, the PCR device system continuously reports the current temperature to the host system while the selected protocol is running in the running phase. In this process, the host system can determine the status of the PCR device and whether the PCR device is operating properly. The PCR protocol used in PCR device consists of a combination of temperature and time since the temperature set in the PCR must be maintained for a specified duration of time. Because PCR is a technique that uses a temperature to replicate and amplify a specific part of DNA, precise temperature control and rapid temperature change are important. Therefore, when grafted with IoT technology, the round-trip time to exchange data is important. Since the PCR chips used in the research are generally operated at about 10°/s, and the temperature can be controlled within 0.5°, the processing period of the control process should be made smaller than 1/20 s (50 ms) [9]. Therefore, the processing cycle about exchanging data should be less than 50 ms. When the IoT technology is incorporated, PCR reports the current temperature of chamber continuously. The user reads the temperature in real time and sends the commands to the PCR to suit the situation. At this time, it is difficult to guarantee the real-time of the temperature control part as the time to exchange data is longer.

When you control the PCR device via the cloud, you should also consider case the problem of network connection state comes up [10]. In the worst case, the network is disconnected during communication. In the case of PCR that transmits and executes the protocol at one time, when the first program is executed, the device is connected to the cloud, and then the user sends the operation command to the cloud. According to the operation commands received from the cloud, the problem does not occur even if the network between the user and the cloud is disconnected while the PCR is executed. In the case of PCR device that transmits the protocol in real time according to the PCR status, if the network is disconnected during operation, there may be a problem in operating the PCR device. After the user sends the operation command via the cloud, the PCR device and the cloud exchange data in when the network is connected. Depending on the situation of the network, the round-trip time for sending and receiving data may become longer or shorter, and we must judge whether the longest delay time is acceptable.

Previous studies have shown that PCR devices can be controlled via the cloud [11]. However, when various data are exchanged, the round-trip time increases as the size of data increases. As a result of the experiment, no problem occurred in the PCR which transmits the protocol at a time. But it was judged that it can cause problems in real-time PCR. Real-time PCR can monitor the amplification process in real time and quantify the amount of amplification [12]. Since Representational State Transfer (REST) Protocol used in previous research is operated on HTTP basis, it is heavy and slow to collect control information and sensor information of small devices. This study uses the messaging technology supported by Firebase to exchange commands and data from PCR devices. Firebase Cloud Messaging (FCM) can transfer up to 4 kB of payload to client apps. Therefore, in this study, the round-trip experiment to measure the time to exchange data with the real-time database using FCM was repeatedly performed. Through this experiment, we confirmed the communication speed and determined the delay time value which can cause the operation of the PCR device.

In “Methods” section, we describe how to construct a device control and real-time monitoring system through the cloud using FCM, and how to measure round trip time to exchange data. Results of the system configuration described in “Methods” section and the result of the round-trip time measurements and conclusions are presented in “Results and discussion” and “Conclusions” sections, respectively.

## Methods

**Fig. 1:**
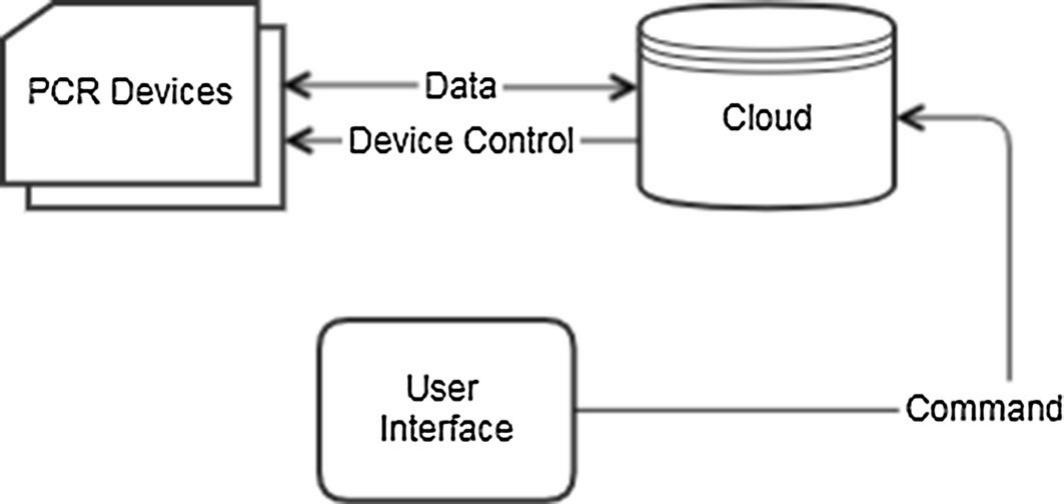
Internet of Things demo flow

Figure 1 shows the flow chart when IoT technology is applied to PCR technology. The user sends a command about the PCR device to the cloud using user interface and controls the PCR device according to the command received by the cloud, and the PCR device continuously transmits the current status to the cloud. The objective of this study is to enable external monitoring and control of PCR by this system. In this study, the current temperature is recorded in the Firebase real time database so that the user can check the current state of the PCR in real time. FCM was used to send commands to the PCR device from the host system. FCM is a cross-platform messaging solution that reliably transmits messages provided by Google. It is a method of transmitting notification messages to client apps via iOS, Android, and Web. In this study, it was developed by setting FCM on the web from the PC. The behavior of the message depends on whether the page is in the foreground state with focus, background state, hidden behind other tabs, or completely closed. In the background state, the notification payload is received in the app’s notification list, and the app handles the data payload only if the user clicks the notification. In the foreground state, the app receives a message object that is served both a notification payload and a data payload. It is difficult for the user to check the status of the PCR device in real time in a background state, hidden behind other tabs, or completely closed. So, we configured display notifications so that users can switch web apps to the foreground if they are in the background state.

**Fig. 2:**
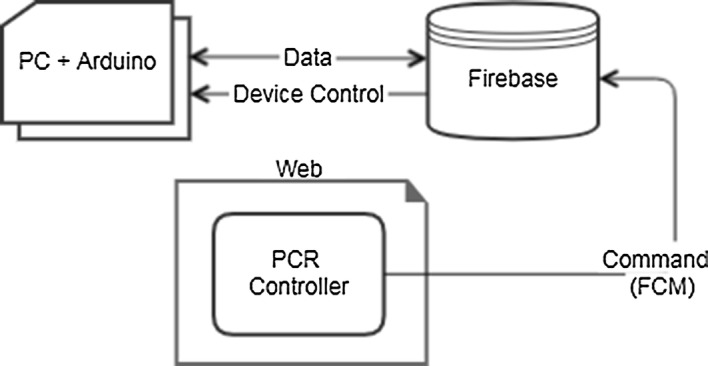
Program work flow

In order to converge the PCR technology with the IoT technology, it takes a lot of time and effort to modify the software of the commercially available PCR device. Currently, this study is an experiment to test the possibility of control via the cloud, so an emulator is used instead of a PCR device. The current program flow chart is shown in Fig. 2. In this study, we used an emulator that imitated a PCR device as described above. Since this device cannot be connected to the network independently, the PC network is used by connecting to the PC separately. As the cloud system, we used Firebase, which is supported by Google. The PCR controller can set operation commands for the PCR device on the web. When the program is run, the PCR device first connects to the cloud and waits for a notification message until the user sends an operation command. The user transmits the operation command such as start and stop to the PCR in the form of a notification message using the FCM through the web. When the PCR device receives a command sent by the user, it operates or stops the device according to the received command and sends data to the cloud. Since the current chamber temperature of the PCR is stored in the cloud in real time, it is possible for the user to check the status of the PCR device from the outside. Check the status of the PCR by continuously reading the cloud from the host system. If the temperature of the PCR is abnormal, notification the user using the FCM. When the operation of the corresponding protocol is completed, the user is notified of the termination, and the PCR device waits until the operation command is received again.

Unlike basic functions such as temperature measurements, the PCR device uses the heater and fan for heating and cooling according to the protocol. The PCR thermocycler is a device enabling precise temperature control and rapid temperature changes to perform PCR technology [13]. In this paper, we use an emulator that mimics the thermocycler of the PCR device. The device implements a thermocycler using a resistor and an NTC thermistor and transmits data to the cloud via the PC.

**Fig. 3:**
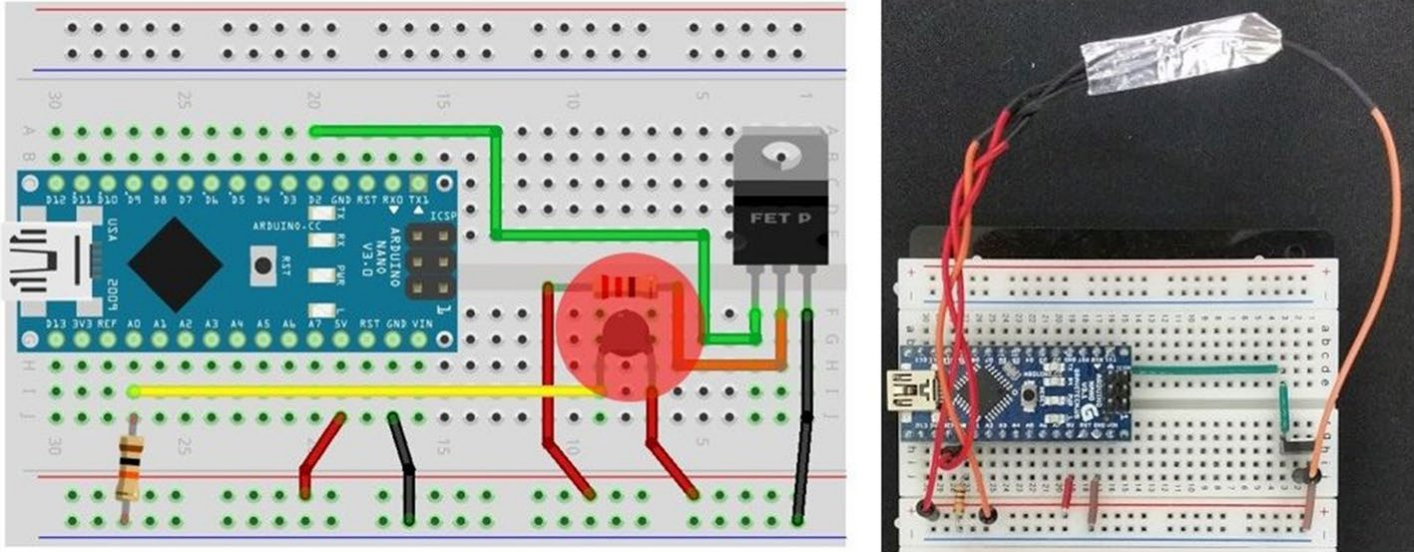
Thermocycler circuit diagram (Arduino)

**Fig. 4:**
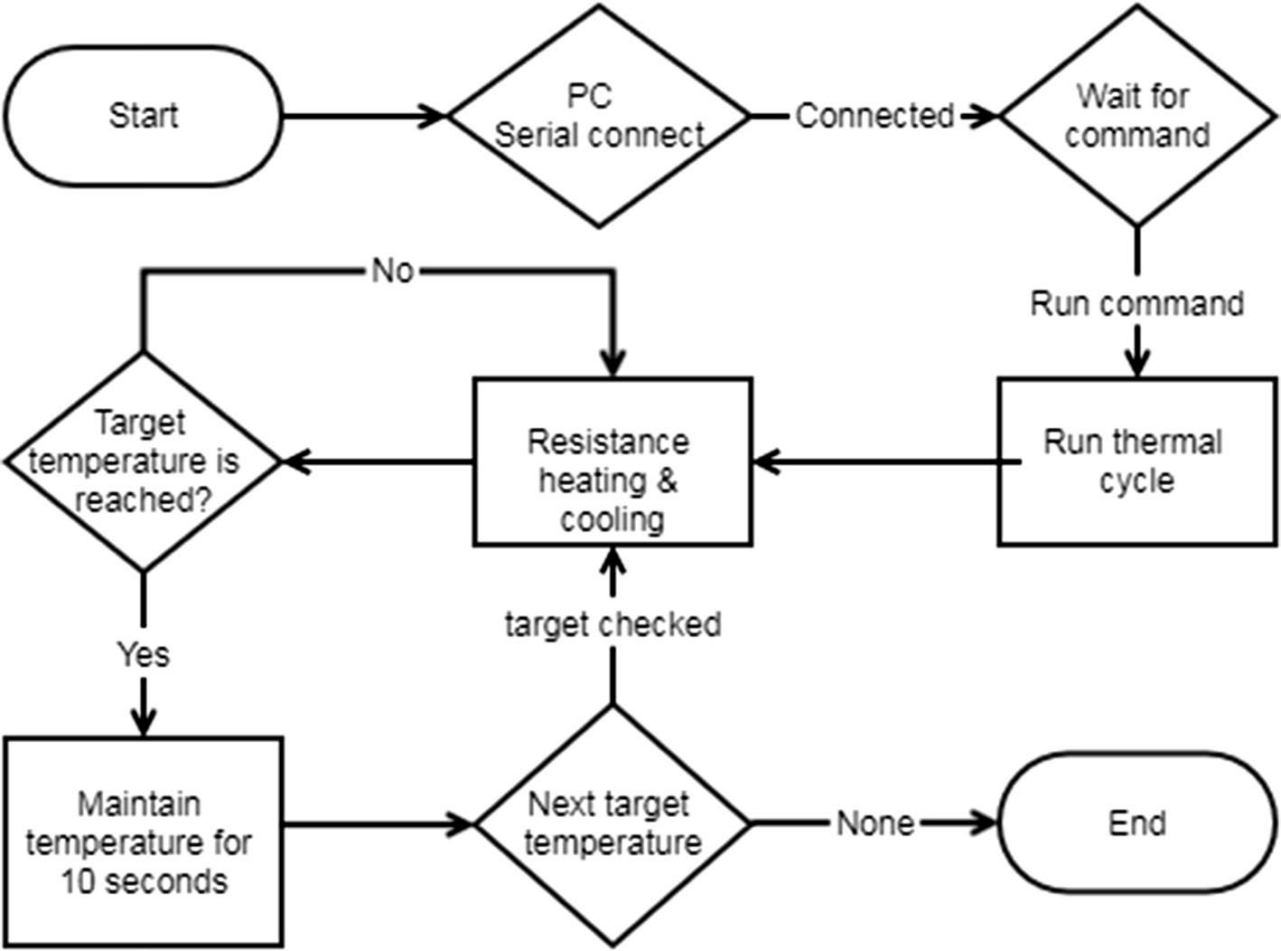
Device (Arduino) work flow chart

The thermistor is attached to the circle shown in the left figure (Fig. 3). In this circuit, the resistor acts as a heater. The resistor is connected to a field effect transistor (FET) and uses Arduino’s pulse width modulation (PWM) to control the temperature. The thermistor measures the temperature as a resistance value, the temperature value can be obtained by converting this resistance value to analog–digital converter (ADC) (Fig. 4).

Arduino shield is the hardware to extend performance by connecting to the board. Arduino, which does not use a shield associated with a network such as an Ethernet shield or a Wi-Fi shield, must rely on the PC to use the network. So, the PC and Arduino allowed the network to be used through serial communication. Arduino first makes a serial communication connection with the PC at the time of execution, and the PC confirming the operation command from the Firebase commands Arduino to operation. In the Arduino, heating and control were performed using resistors, and the NTC thermistor acted as a temperature controller. To reach the target temperature, the resistance is heated or naturally cooled, and when the target temperature is reached, the target temperature is maintained for the set period of time using on/off control. When the set protocol is completed, the Arduino program is terminated. In addition, it performs the ability to the PC sends the data received from the device to the cloud, or sends the data received from the cloud to the device.

When monitoring or controlling PCR from outside by combining cloud computing technology, the communication speed between client and cloud environment is an important issue. Previous studies have confirmed that PCR devices can be controlled via the cloud using the REST API. The study found that the larger the size of data between client and PCR devices, the longer the round-trip time was extended. In this study, we conducted experiments to measure the time of data exchange using cloud messaging supported by Firebase. Through this experiment, we determined whether the longest delay time could cause problems in the operation of PCR.

## Results and discussion

The PCR controller has been established on the web to allow the user to send operation commands to the device from outside. The command sent to the devices sets as ‘START’ or ‘STOP’ commands, which is sent to the cloud so that the devices can be operated.

**Fig. 5:**
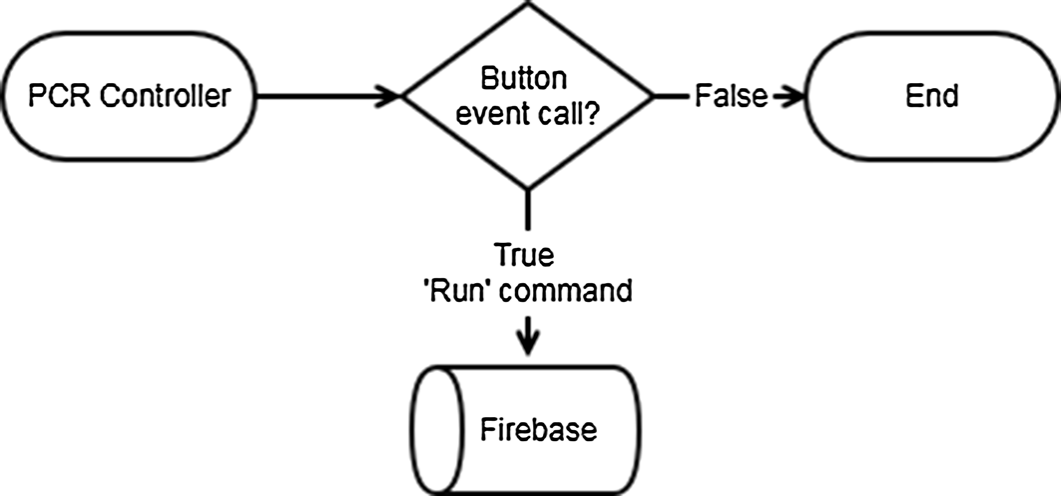
The operation to start the PCR

**Fig. 6:**
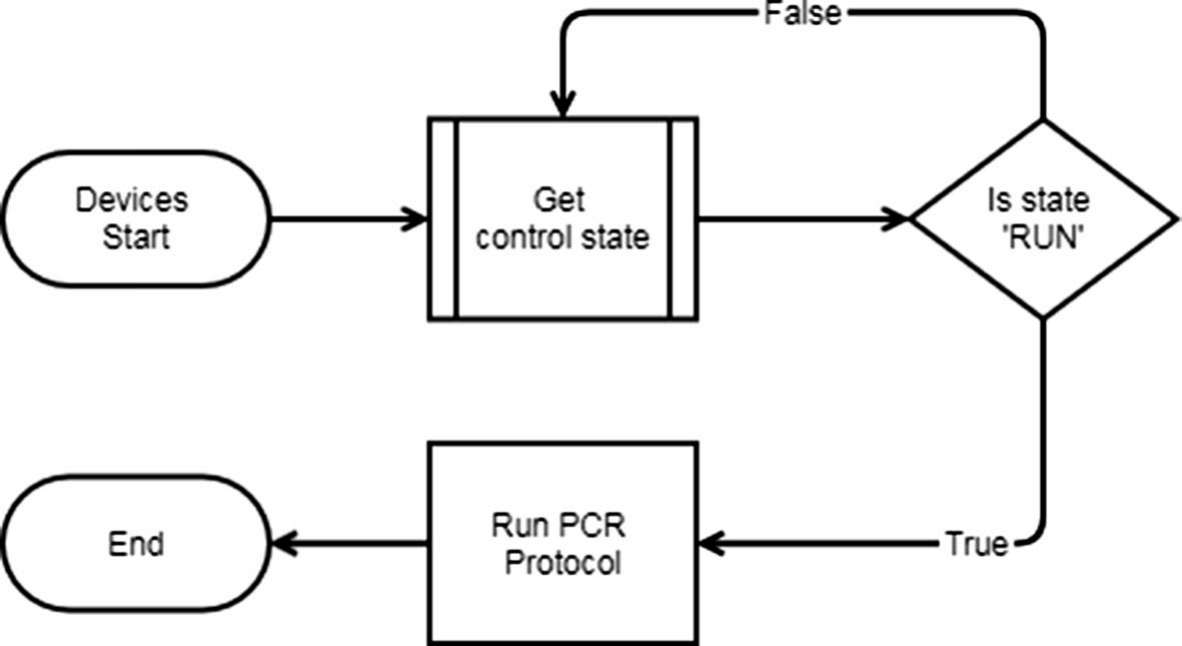
Device flow chart

The token id is given to each user in Firebase at the start of the initial program (Fig. 5). User can receive FCM through a given token id. The token id contains authentication-related information along with data identifying the user. When a user or device successfully logs in, Firebase creates an appropriate identity token that uniquely identifies them and allows access to multiple resources, such as the Firebase real-time database and cloud storage. When ‘RUN command’ is called through button click event on web page, ‘RUN’ command is recorded in control related table of Firebase. The device continuously checks the control status from the Firebase, then confirms the ‘RUN’ command and proceeds with the specified operation (Fig. 6). When all the operations are completed, the devices indicate ‘STOP’ in the control related table of the Firebase and at the same time the user can receive a notification message informing the status of the device.

At this point, a notification message notifying the user about the status of the PCR or a command message sent to the cloud by the user is transmitted via the FCM. Therefore, we conducted an experiment to determine the delay time of the FCM and whether how long the delay time was acceptable.

**Fig. 7:**
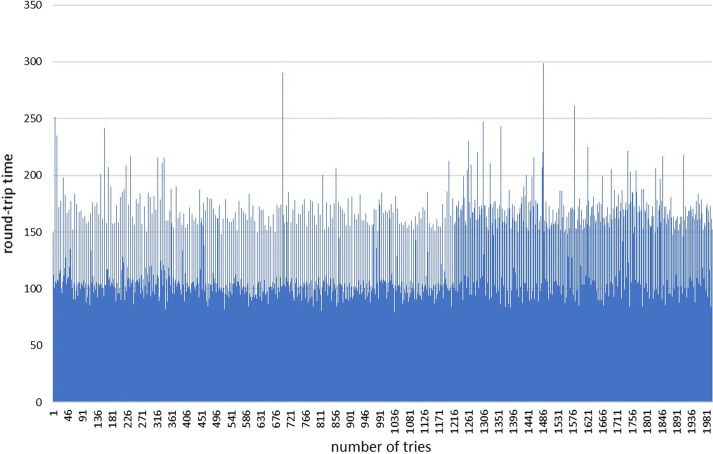
Data round-trip time

We measured of the time it takes to send a message to the client via the FCM. The x-axis represents the number of times the message is transmitted, and the y-axis represents the communication rate in milliseconds (Fig. 7). As a result, the minimum communication speed was measured at 77.35 ms and the average communication speed was 150.33 ms. As mentioned above, the PCR chip of the PCR device used in this study is judged to be able to control the temperature when the time of data exchange is less than 50 ms. However, the average speed of the experiment is 150.33 ms, which is slow. The PCR chip used in this study requires 20 control events per second. However, the experimental results showed that the speed of communication is approximately three times slower than the speed of 50 ms we set as the reference point. It is possible to control the PCR device via the cloud up to the system controlling 2/3°/s. However, in the case of currently used PCR devices, it is impossible to control 10°/s via the cloud. However, it is possible for the user to monitor the status in real time with an average time of 150 ms.

## Conclusions

In this study, we conducted experiments to search if PCR device and IoT technology could be convergence to minimize the inconvenience that users may feel in controlling existing PCR instruments. In this experiment, we sent a status command message to the user to start or stop the PCR device from the outside, and the user can be received a notification message about the status of the PCR device using FCM. As mentioned above, when the IoT technology is applied, the control over the cloud is influenced by the communication state of the network. So, we measured the time to exchange FCM. We have experimented to find the delay time of the FCM when there is no problem in operating the PCR device.

The time the user externally monitors can be delayed by an average of 150 ms. This is a short time for monitoring, so there is no problem for the user to monitor the PCR device. However, it was considered impossible to control the temperature during the PCR control process. As mentioned earlier, the PCR device used in this study may cause problems because the processing time is required to be of approximately 50 ms. Thus, it is not possible for PCR devices that require 20 control events per second, such as the PCR device used in this study. However, in case of a system that can control 2/3°/s, it is possible to control via the cloud.

Currently, the proposed system is a system that sends an operation command to the cloud from the GUI and the device continues to read the command part of the cloud and operate the device. However, since the system control through the GUI can cause problems, it is necessary to have a system that can perform from device to control.

## Abbreviations

PCR: polymerase chain reaction
FCM: Firebase Cloud Messaging
REST: Representational State Transfer
IoT: Internet of Things
HTTP: Hyper Text Transfer Protocol
FET: field effect transistor
PWM: pulse width modulation
ADC: analog–digital converter
NTC: negative temperature coefficient thermistor
GUI: graphical user interface
